# Time-Resolved Chemical Bonding Structure Evolution by Direct-Dynamics Chemical Simulations

**DOI:** 10.1021/acs.jpclett.4c03010

**Published:** 2024-11-28

**Authors:** Mario Piris, Xabier Lopez, Jesus M. Ugalde

**Affiliations:** † Donostia International Physics Center (DIPC) & Kimika Fakultatea, Euskal Herriko Unibertsitatea (UPV/EHU), 20018 Donostia, Euskadi, Spain; ‡ Basque Foundation for Science (IKERBASQUE), 48009 Bilbao, Euskadi, Spain

## Abstract

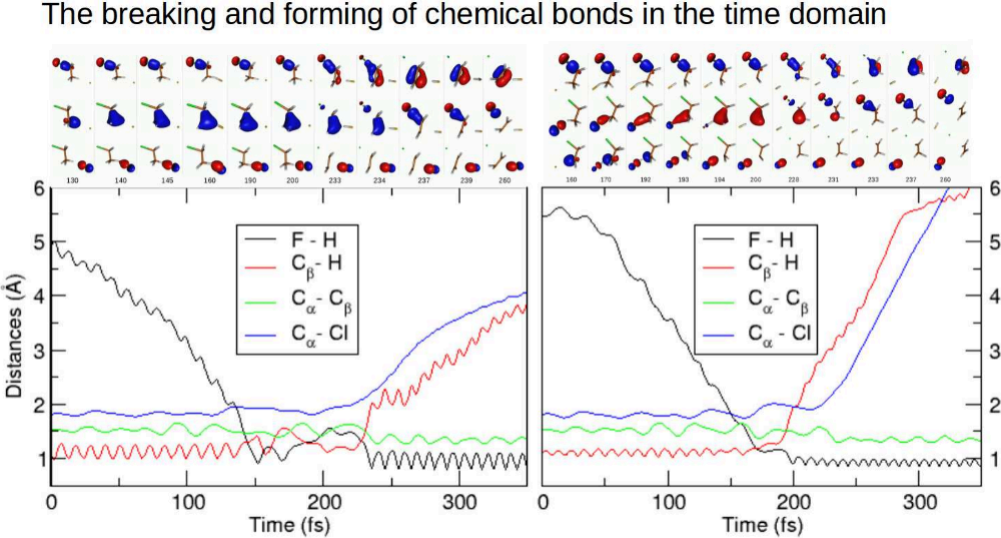

Direct-dynamics simulations monitor atomic nuclei trajectories during chemical reactions, where chemical bonds are broken and new ones are formed. While they provide valuable information about the ongoing nuclear dynamics, the evolution of the chemical bonds is customarily overlooked, thus, hindering key information about the reaction mechanism. Here we examine the evolution of the chemical bonds for the three main mechanisms of the F^–^ + CH_3_CH_2_Cl reaction using quasi-classical trajectories for the nuclei, and global natural orbitals for the electrons. Key findings include (i) bimolecular nucleophilic substitution (S_N_2) resembles a one-step bond breaking and formation process; (ii) the elimination mechanisms (*syn*- and *anti*-E2) feature a sequential two-step process of proton abstraction and Cl^–^ elimination; and (iii) the *anti*-E2 mechanism is slower, exhibits rebound effects, and gets activated by specific vibrational modes. This study highlights the importance of correctly describing and thoroughly analyzing the dynamical evolution of chemical bonds for chemical reaction mechanistic studies.

Chemical reactions constitute (complex) rate processes that involve the concerted time evolution of both the nuclei and the electrons of the molecules engaged in the reaction from reactants to products. From a quantum chemistry perspective, the adiabatic time evolution of the nuclei is customarily carried out in the classical domain, and the electrons are assumed to respond quickly enough to the nuclei’s evolution as to justify taking them under the auspices of the Born–Oppenheimer (BO) approximation.^1^ Nonadiabatic time evolution dynamics, which requires the consideration of transitions between different electronic states, can be conveniently handled by potential surface hopping methods.^2^

Consequently, the study of chemical rate processes consists of (i) selecting the appropriate quantum mechanical method to treat the electrons; (ii) solving the corresponding Schrödinger equation for the energy and estimating its gradient at the position of each nucleus; (iii) making judicious choices of the initial conditions for the nuclei in the ensemble of trajectories to be propagated in time; (iv) numerically integrating the classical equations-of-motion to determine the time evolution of each nucleus in each calculated trajectory; and (v) transforming the trajectories’ final nuclei coordinates and momenta into properties that may be compared with experimental results. As indicated elsewhere,^3^ the properties at stake include bond lengths and angles, vibrational, rotational, and translation energies, quantum numbers for the vibrational and rotational degrees of freedom, the amount of energy in individual molecular degrees of freedom, and scattering angles. These are all properties that naturally derive from the positions and momenta of the nuclei but omit further considerations of the chemical bonding structure evolution along the trajectory. The latter is simply not addressed by most chemical dynamics simulation studies.

However, a chemical reaction is conceived as a process in which chemical components are transformed to other chemical components. Since chemists identify components by their chemical bonds, i.e., their electronic structure, the evolution of these bonds during the reaction carries significant chemical information encoded in the concept of the reaction mechanism. This refers to the time-ordered sequence of forming and/or breaking chemical bonds, along with the characterization of all transient chemical components throughout the trajectory from reactants to products.^4^

Electronic structure calculations enter into chemical dynamics simulations in steps (*i*) and (*ii*) mentioned above. However, these are demanding calculations aimed at not only dealing with static considerations of structure but also confronting the dynamics of the replacements of some chemical bonds into newly formed ones that takes place in the course of chemical reactions. A word of caution about their appropriateness is in order for most such calculations do not produce chemically acceptable descriptions of the whole evolution of the making and/or breaking of chemical bonds.^5^

Accordingly, we have employed the recently introduced Global Natural Orbital Functional (GNOF)^6^ to evaluate the BO energies, as previous studies^7,8^ have demonstrated that GNOF yields a favorable balance between static and dynamic electron correlations, resulting in chemically informative descriptions and accurate total energies throughout the entire range of internuclear coordinates, from stable structures to their dissociated asymptotic limits. This is a crucial feature for the present investigation, which focuses on the analysis of chemical bonds as they form and break. Additional information on GNOF and its analytic gradients can be found in Appendix.

This paper aims to communicate the feasibility of quasi-classical trajectory (QCT) direct-dynamics simulations, supported by GNOF calculations of the electronic structure, to determine the time evolution of the making and breaking of chemical bonds in reactive processes, which enriches the already available information about nuclear dynamics.^9^ We have selected for such a purpose the reaction between the fluoride anion and ethyl chloride, an extensively studied canonical case.^10−18^ Theoretical simulations conducted to date, along with their comparison to experimental data, have successfully addressed energy- and angle-differential reactive scattering cross sections and have effectively distinguished between the competing nucleophilic substitution (S_N_2) and base-induced elimination (*syn*- and *anti*-E2) reactions. Additionally, it has been established that the reaction of F^–^ + CH_3_CH_2_Cl is predominantly governed by direct dynamics, except at low collision energies. The *anti*-E2 mechanism has emerged as the dominant mechanism, although the S_N_2 and *syn*-E2 mechanisms gain relevance as the collision energy increases.

It is worth noting that the present study aims not at replicating already explored aspects of the title reaction but at providing a better understanding of the operating mechanisms. This will be achieved by a thorough analysis of the time evolution of the chemically active bonds along selected trajectories. Thus, based on the previous results,^10−18^ we will choose impact parameters and collision energies such that we can monitor reactive trajectories and observe the time evolution of the chemically active natural orbitals and, consequently, the evolution of the chemical bonding structure along the selected reactive trajectories.

Earlier high-level electronic structure calculations^19^ have identified three primary reaction channels: (i) bimolecular base-induced elimination, which proceeds via both the *anti*-E2 and *syn*-E2 pathways, resulting in the formation of CH_2_=CH_2_ + HF + Cl^–^, and (ii) bimolecular nucleophilic substitution (S_N_2), where the incoming F^–^ attacks the C_α_ carbon atom from either the back or front side relative to Cl, producing CH_3_CH_2_F + Cl^–^. However, the front-side attack has a too-high energy barrier of ∼1.3 eV. Accordingly, we adjusted the impact parameter and the direction of the initial velocity vector of the F^–^ anion to favor the three primary mechanisms: (back-side) S_N_2, *anti*-E2, and *syn*-E2.

All calculations were performed using the molecular dynamics module integrated into the DoNOF computer program,^20^ which employs Beeman’s algorithm^21^ to numerically integrate the classical Newton equations-of-motion for the nuclear coordinates, with forces determined on-the-fly as the gradient of the total BO energy at each nuclear position.

The initial conditions for Beeman’s numerical integration are selected to represent the quantum mechanical vibrational and rotational energy levels of the reactants in their ground state. Consequently, our calculations should be classified as quasi-classical trajectory (QCT) BO direct-dynamics simulations.^3^

The equilibrium structure of ethyl chloride was determined via a full optimization using the GNOF with the cc-pVDZ basis set.^22,23^ The molecule was positioned at the center of the laboratory coordinate system, and the initial nuclear momenta were adjusted according to the equilibrium molecular normal vibrational modes.

Previous simulations have identified multiple direct and indirect mechanisms of S_N_2/E2 reactions. It has been established that both reactions transition from a highly indirect character to a direct one as collision energy increases. Schematic potential energy diagrams for the three relevant channels depict similar double-well potential energy surfaces (PESs), with a transition state connecting the reactant and product complexes.^15^ The corresponding submerged barriers are extremely close, highlighting the pivotal role of dynamics in the competition between *anti*-E2, *syn*-E2, and S_N_2 pathways. Consequently, we selected a kinetic energy of *E*_k_ = 0.2 eV in the laboratory coordinate system for F^–^, which corresponds to a low collision energy of 0.154 eV in the center-of-mass coordinate system, where indirect mechanisms dominate.

The initial separation between the fluoride anion and the center-of-mass of ethyl chloride was set at 6 Å, and each trajectory was integrated until the separation between the final fragments exceeded 6 Å. A time step of 0.1 fs was utilized. The potential energy profiles for the three studied reactive trajectories with F^–^ translational energy *E*_k_ = 0.2 eV and CH_3_CH_2_Cl in the ground state can be seen in Figure 1, while a movie of these trajectories is available in the Supporting Information. Based on these data, we can conclude that the reactive collisions occur roughly within the time range of 130–270 fs.

**Figure 1:**
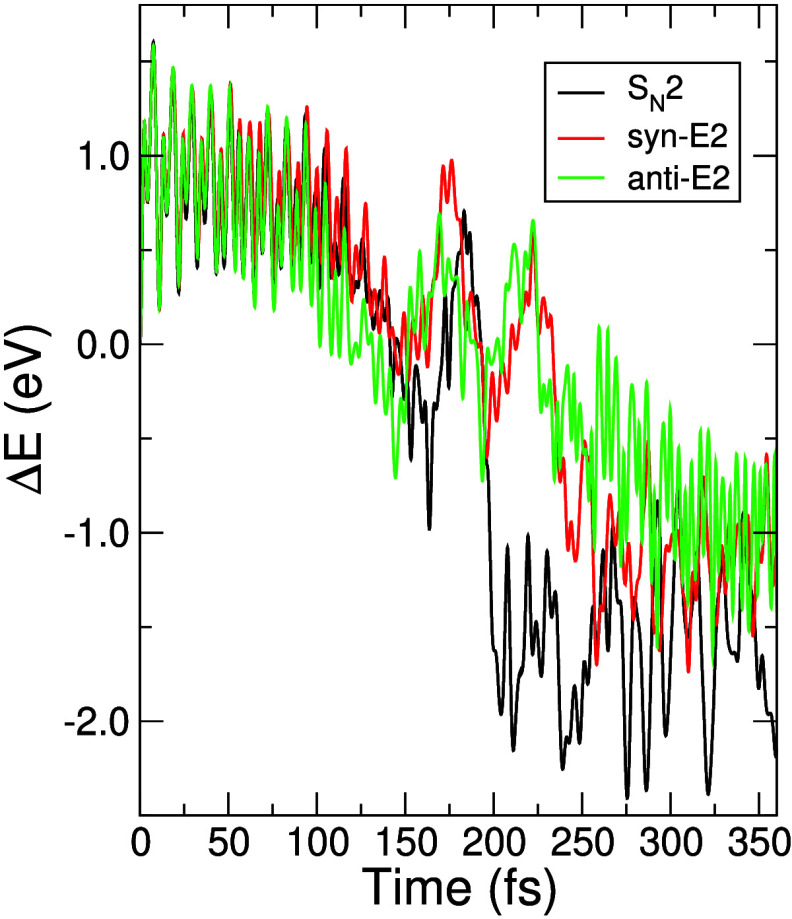
Potential energy profiles of reactive trajectories relative to the energy of the reactants, for F^–^ with a translational energy of 0.2 eV and CH_3_CH_2_Cl in the ground state. The time step used was 0.1 fs.

The fast fluctuations of the total potential energy for the three mentioned pathways, shown in Figure 1 at both short and long times, reflect the energy oscillations of the quasi-harmonic vibrational modes of the reactants and products, respectively. Observe that, at intermediate times, the oscillation pattern markedly differs from quasi-harmonic, indicative of chemical bonding rearrangement being taking place. The precise nature of the occurring rearrangement for each pathway will be discussed below by inspecting the time evolution of the relevant bonding natural orbitals.

The chemical-bonding structure evolution is reflected in a handful of valence natural orbitals. These orbitals, hereafter termed the “chemically active orbitals”, will be discussed for each of three main reaction mechanisms. Full movies of both the nuclear dynamics and the dynamical evolution of the chemically active orbitals may be found in the Supporting Information video files.

## S_N_2 Mechanism

**Figure 2:**
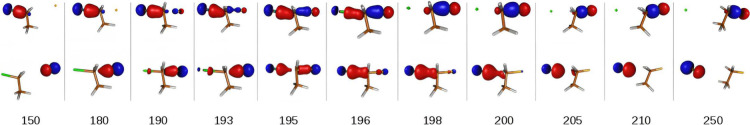
Time evolution of the two chemically active natural orbitals for the S_N_2 mechanism. The bottom line indicates the time stamps (femtoseconds) of the selected snapshots. Contour value = 0.1 au. The top row natural-orbitals’ snapshots show the adiabatic evolution of the σ(Cl–C_α_) bond to the σ(C_α_–F) bond. The bottom row shows the adiabatic evolution of the F^–^(2p_*z*_) orbital to the Cl^–^(3p_*z*_) orbital. The tiny orange and green dots represent the positions of F^–^ and Cl^–^, respectively.

Figure 2 shows the time evolution of the chemically active natural orbitals of the S_N_2 mechanism of the F^–^ + CH_3_CH_2_Cl → Cl^–^ + CH_3_CH_2_F reaction.

Notice that these two orbitals describe the breaking of the Cl–C_α_ bond and the formation of the C_α_–F bond. Inspection of their time evolution reveals that these two processes, Cl–C_α_ bond breaking and C_α_–F bond formation, occur synchronously in a single step, which is completed in ∼100 fs time. Ocular inspection of the frames shown in Figure 2 suggests that the transition state has a lifetime of ∼8 fs. Notice that both the σ(Cl–C_α_) and σ(C_α_–F) bonds begin their breaking and forming processes, respectively, at *t* = 190 fs and finish at *t* = 198 fs.

## *syn*-E2 Mechanism

**Figure 3:**
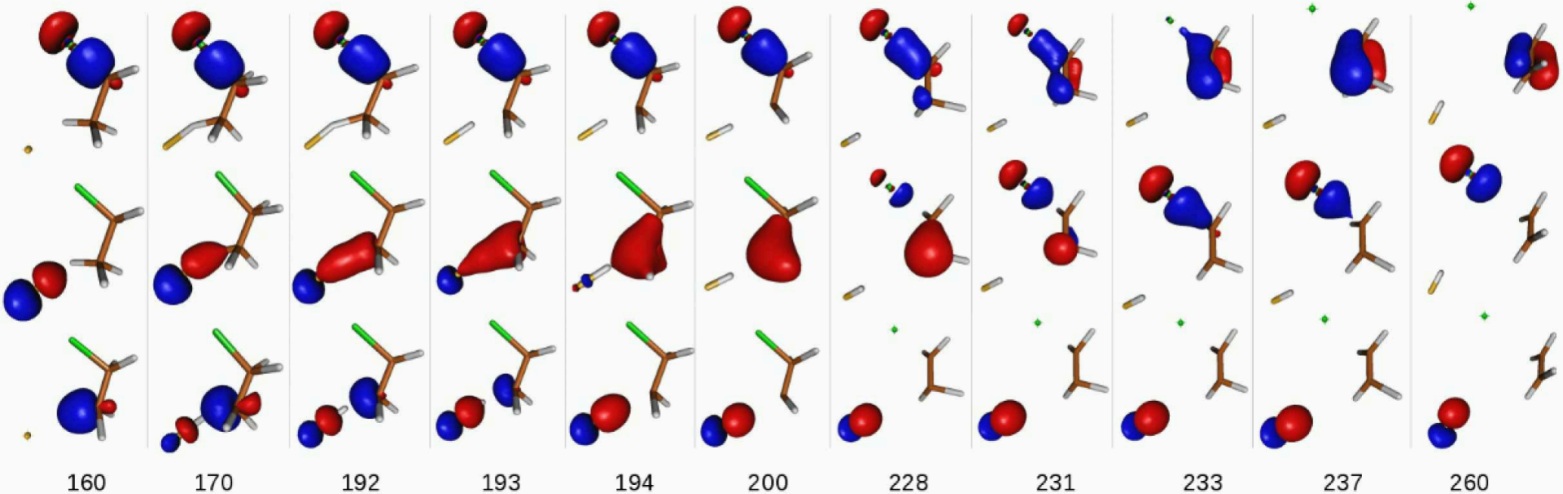
Time evolution of the three chemically active natural orbitals for the *syn*-2 mechanism. The bottom line indicates the time stamps (fs) of the selected snapshots. Contour value = 0.1 au. The top row natural-orbitals’ snapshots show the adiabatic evolution of the σ(Cl–C_α_) bond to the π(C_α_–C_β_) bond. The middle row shows the adiabatic evolution of the F^–^(2p_*z*_) orbital to the Cl^–^(3p_*z*_) orbital, and the bottom row shows the adiabatic evolution of the σ(H–C_β_) bond to the σ(F–H) bond. The tiny orange and green dots represent the positions of F^–^ and Cl^–^, respectively. See text for further details.

Figure 3 shows the orbital time evolution of the chemically active natural orbitals of the *syn*-E2 mechanism for the F^–^ + CH_3_CH_2_Cl → FH + C_2_H_4_ + Cl^–^ reaction.

Here we can see the initial proton abstraction from C_β_ by the incident F^–^ anion as reflected in the time evolution of the two natural orbitals shown in the bottom rows of Figure 3, between *t* = 160 and 194 fs. Thus, one can clearly observe the formation of the σ(F–H) bond (as seen in the bottom row frames) and the emergence of a carbanion at C_β_, illustrated in the middle row frames. The end result is the formation of a transient hydrogen fluorine “stabilized” chlorinated carbanion depicted in the frame corresponding to *t* = 194 fs, where these three salient elements of its electronic structure are neatly shown. Namely, the top frame shows the σ(Cl–C_α_) bond, the middle frame shows the ClH_2_C_α_–C_β_^⊖^H_2_ carbanion, and the bottom frame shows the σ(H–F) bond.

From this “critical point” onward, the evolution of the electron density, i.e., the chemical bonding structure, moves on from the vicinity of C_β_ to C_α_. Indeed, inspection of frames *t* = 200 fs −237 fs reveals the synchronous formation of the new π(C–C) bond and the detachment of the Cl^–^ anion upon breaking of the σ(Cl–C_α_) bond. This process takes ∼43 fs.

The main observation here is the sequential occurrence of two processes. The former constitutes the proton abstraction by F^–^ from the H–C_β_ σ-bond and the generation of a carbanion at C_β_ by retention of the bonding electron pair of the σ(H–C_β_) bond at the C_β_ carbon atom. The latter process consists of the rearrangement of the bonding structure around the C_α_ carbon to yield a newly formed π(C–C) bond and a chlorine anion which takes the electron pair of the σ(Cl–C_α_) bond as it breaks apart.

These two processes are decoupled in both time- and real-space coordinate domains. They occur at different reaction times and in different locations. All in all, the above findings suggest that the *syn*-E2 mechanism resembles a two-step process where bond breaking and formation processes do not take place simultaneously but sequentially, opposite to S_N_2 (vide supra).

## *anti*-E2 Mechanism

**Figure 4:**
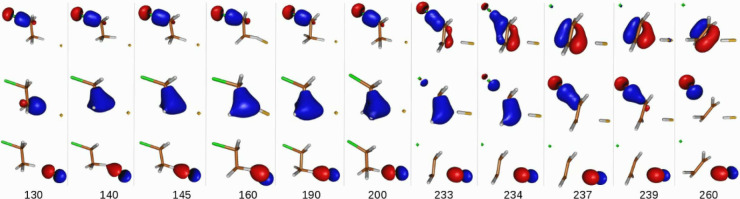
Time evolution of the three chemically active natural orbitals for the *anti*-E2 mechanism. The bottom line indicates the time stamps (fs) of the selected snapshots. Contour value = 0.1 au. The top row natural-orbitals’ snapshots show the adiabatic evolution of the σ(Cl–C_α_) bond to the π(C_α_–C_β_) bond. The middle row shows the adiabatic evolution of the σ(H–C_β_) bond to the Cl^–^(3p_*z*_) orbital, and the bottom row shows the adiabatic evolution of the F^–^(2p_*z*_) orbital to the σ(F–H) bond. The tiny orange and green dots represent the positions of F^–^ and Cl^–^, respectively. See text for further details.

The inspection of the chemically active natural orbitals of the *anti*-E2 mechanism, whose time evolution is shown in Figure 4, highlights not only some similarities with *syn*-E2 but also a number of profound and consequential differences, which will be discussed below.

First, we observe that the reaction “starts” earlier than *syn*-E2. The interaction between the F^–^ fluorine anion and the proton of σ(H–C_β_) is noticeable in the bottom row frame at *t* = 140 fs, whereas the onset for this interaction is 170 fs for *syn*-E2.

Second, it is observed that the proton abstraction takes longer to complete. Namely, the “critical point” is arrived at *t* = 233 fs, which is to be compared with *t* = 194 fs for *syn*-E2. Further inspection of the evolution of the natural orbital describing the forming σ(H–F) bond (see bottom row frames from *t* = 130 fs to *t* = 233 fs) shows a *rebound* effect, meaning that such an σ(H–F) orbital reverses its development from *t* = 145 fs to *t* = 190 fs (see Figure S1 in the Supporting Information). Subsequently, from *t* = 190 fs onward the σ(H–F) bond formation resumes, and at *t* = 233 fs it gets fully formed and the C_β_–H is broken. This is very suggestive of the key role played by the H–C_β_ stretching vibrational mode-specific excitation for this mechanism (vide infra).

Third, similarly to the *syn*-E2 mechanism, the “critical point” marks the point for the evolution of the chemical bonding structure evolution to move on from the vicinity of C_β_ to C_α_. From this critical point onward, the synchronous formation of the π(C–C) bond, at the expense of the delocalization of the C_β_ carbanion’s electron pair over both carbon atoms, which necessarily turns out into a π-symmetry orbital due to the required orthogonality to the already existing σ-symmetry C–C bonding orbital, and the detachment of the Cl^–^ chlorine anion, at the expense of the σ(Cl–C_α_) bond breaking, take place in a short time of ∼6 fs. The difference is that now the “critical point” is arrived at *t* = 233 fs, ∼40 fs later than in *syn*-E2. This observation suggests that *anti*-E2 shows a late barrier (vide infra).

## Outline

Since the mechanism of a chemical reaction refers to the order in which chemical bonds are made and/or broken,^4^ it follows naturally that the time evolution of the molecular natural orbitals is the proper “place” to look for chemically insightful descriptions of reaction mechanisms.

The present study reports on the adiabatically relaxed molecular natural orbitals along selected trajectories for the canonical bimolecular (i) nucleophilic substitution and (ii) base-induced elimination reaction mechanisms of F^–^ + CH_3_CH_2_Cl, revealing how these analyses yield significant chemical insight into both competing reaction mechanisms.

Our study takes advantage of the BO *ab initio* molecular dynamics where the adiabatic relaxation of the electronic structure is carried out by GNOF calculations. Thus, the nuclei are propagated following the classical equations of motion, and at each time step, the quantum mechanical electronic structure problem at the clamped nuclear positions is addressed using GNOF, which has been reported to be chemically accurate at describing molecular bond formation and breaking processes. Consequently, our procedure provides a detailed and reliable chemical description of the time evolution of the chemical bonding structure of the species involved in the reaction for each selected mechanism.

We have shown that the S_N_2 nucleophilic substitution proceeds in one single step with the formation of the σ(F–C_α_) chemical bond and the breaking of the σ(C_α_–Cl) bond taking place synchronously. The chemical environment around the C_β_ atom remains a spectator during the completion of the halogen substitution.

The *syn*-E2 and the *anti*-E2 elimination mechanisms are substantially more complex because chemical action takes place on the two carbon atoms of CH_3_CH_2_Cl in a concerted but sequential way. The time-ordered chemical events include the abstraction of a proton from a σ(H–C_β_) bond by the incoming F^–^ anion, and the subsequent formation of a transient carbanion at *C*_β_. During the completion of this first step, the C_α_ moiety remains expectant. Next, the second step consists of the delocalization of the electron pair of the C_β_ carbanion into the C_α_, to form a π-type covalent bond between the two carbon atoms, and the Cl^–^ detachment from the σ(Cl–C_α_) bond. We observe, therefore, two sequential steps taking place in this order at the *C*_β_ and in the C_α_ atoms, which feature two decoupled bond breaking/formation processes occurring in different time- and real-space coordinate domains, neatly divided by the barrier epitomized by the hydrogen fluorine “stabilized” chlorinated carbanion transient structure.

Furthermore, we observe that the proton abstraction by the incoming F^–^ anion from the σ(H–C_β_) bond takes a longer time in *anti*-E2 (233 fs) than in *syn*-E2 (194 fs) and exhibits “rebound” effects in the former, in the sense that the proton moves back and forth between C_β_ and F^–^. This is very suggestive that vibrationally exciting the H–C_β_ stretching mode should enhance the proton abstraction and, eventually, should lead to *anti*-E2 dominance over both *syn*-E2 and S_N_2 mechanisms, as suggested elsewhere.^24,25^

The second important implication of the longer time taken by the initial proton abstraction in *anti*-E2, is that the second step, i.e., the formation of the π(C–C) bond and detachment of Cl^–^, takes place at later times. Namely, the “barrier” for the transition from the first step to the second comes at later times in the *anti*-E2 bond relative to the S_N_2 mechanism. This is in accordance with the extension of the Polanyi rules^26^ to polyatomic reactive processes.^25^ Namely, increasing collision energy less efficiently activates late-barrier mechanisms than early barrier mechanisms and vice versa. Indeed, the experiments^15^ of Meyer et al. convincingly show that for the herein studied reaction, *anti*-E2 ceases to be the most important mechanism at increased collision energies.

## Appendix

Let us consider a mixed singlet state of N spin-paired electrons, specifically 44 in the F^–^ + CH_3_CH_2_Cl reaction. The GNOF is an electron-pairing based approach,^27^ so the orbital space Ω is divided into *N*/2 mutually disjoint subspaces Ω_*g*_. Each orbital exclusively belongs to one Ω_*g*_, housing one strongly double-occupied orbital *g* and *N*_*g*_ weakly double-occupied orbitals. This partitioning ensures that the sum of the occupation numbers equals 2 for each subspace, with the trace of the one-particle reduced density matrix equating the total number of electrons.

The reconstruction functional for the two-particle reduced density matrix in terms of the occupation numbers leads to GNOF, as illustrated by the equation

1

The intrapair component is formed by summing the energies of electron pairs, specifically,

2

where *H*_*pp*_ are the diagonal one-electron matrix elements of the kinetic energy and external potential operators, whereas *L*_*pq*_ = ⟨*pp*|*qq*⟩ are the exchange-time-inversion integrals.^28^ The matrix elements Π(*n*_*q*_, *n*_*p*_) = *c*(*n*_*q*_) *c*(*n*_*p*_), where *c*(*n*_*p*_) is defined by the square root of the occupation numbers according to the following rule:

3

that is, the phase factor of *c*(*n*_*p*_) is chosen to be +1 for the strongly occupied orbital of a given subspace Ω_*g*_, and −1 otherwise. The inter-subspace Hartree–Fock (HF) term is

4

where *J*_*pq*_ = ⟨*pq*|*pq*⟩ and *K*_*pq*_ = ⟨*pq*|*qp*⟩ are the Coulomb and exchange integrals, respectively. The prime in the summation indicates that only the inter-subspace terms are taken into account. The inter-subspace static component is written as

5

where  with the hole *h*_*p*_ = 1–*n*_*p*_. Finally, the inter-subspace dynamic energy is

6

In eqs 5 and 6, Ω^*b*^ denotes the subspace composed of orbitals below the level *N*/2, whereas *n*_*p*_^*d*^ is the dynamic part of the occupation number *n*_*p*_ in accordance with the Pulay criterion that establishes an occupancy deviation of approximately 0.01 with respect to 1 or 0 for a natural orbital to contribute to the dynamic correlation.

In the BO approximation, the total energy is cast as *E* = *E*_*el*_ + *E*_*nuc*_, where *E*_*el*_ represents the electronic energy calculated using (1), and *E*_*nuc*_ is the nuclear energy . Here, *Z*_*A*_ denotes the atomic number of nucleus *A*, and *R*_*AB*_ is the distance between nuclei *A* and *B*.

Considering that all natural orbitals are expanded in a fixed atomic basis set, the derivative of the total energy with respect to the coordinate *x* of nucleus *A* is given by^29^

7

where Γ_*μυ*_ and *D*_*μηυδ*_ are the GNOF one-particle and two-particle reduced density matrices, respectively, *S*_*μυ*_ = ⟨μ|υ⟩ is the overlap matrix, and λ_*μυ*_ are obtained from the reduced density matrices, all in the atomic orbital representation. The derivatives in eq 7 have an explicit dependence on the nuclear coordinate *x*_*A*_, so the force acting on each nucleus *A* (**F**_*A*_ = −∇_*A*_*E*) can be obtained by a single static evaluation at each time step for the fixed nuclear positions at that instant.

## Supporting Information Available

S_N_2 reaction (MP4)
*syn*-E2 reaction (MP4)
*anti*-E2 reaction (MP4)
Figure of interatomic distances versus time (PDF)
Transparent Peer Review report available (PDF)
