# Supplementary material for: Time-Resolved Chemical Bonding Structure Evolution by Direct-Dynamics Chemical Simulations
Source: J Phys Chem Lett. 2024 Nov 28;15(49):12138–43. doi: 10.1021/acs.jpclett.4c03010 (PMC11648084; doi:10.1021/acs.jpclett.4c03010)
Supplement: Supplementary file 4 — jz4c03010_si_004.pdf [file jz4c03010_si_004.pdf]

Time-resolved chemical bonding structure evolution by direct-dynamics chemical simulations – Supplementary Material –

Mario Piris,[*,†,‡] Xabier Lopez,[†] and Jesus M. Ugalde[†]

†*Donostia International Physics Center (DIPC) & Kimika Fakultatea, Euskal Herriko Unibertsitatea (UPV/EHU), Donostia, Euskadi, Spain*

‡*Basque Foundation for Science (IKERBASQUE), Bilbao, Euskadi, Spain*

E-mail: mario.piris@ehu.eus

suppmat-sn2.mp4 file

Movie mp4-formatted file showing the nuclear dynamics, and the dynamical evolution of the chemically active orbitals for the bimolecular substitution S_N2 mechanism.

suppmat-e2-syn.mp4

Movie mp4-formatted file showing the nuclear dynamics, and the dynamical evolution of the chemically active orbitals for the bimolecular base-induced elimination *syn*–E2 mechanism.

suppmat-e2-anti.mp4

Movie mp4-formatted file showing the nuclear dynamics, and the dynamical evolution of the chemically active orbitals for the bimolecular base-induced elimination *anti*–E2 mechanism.

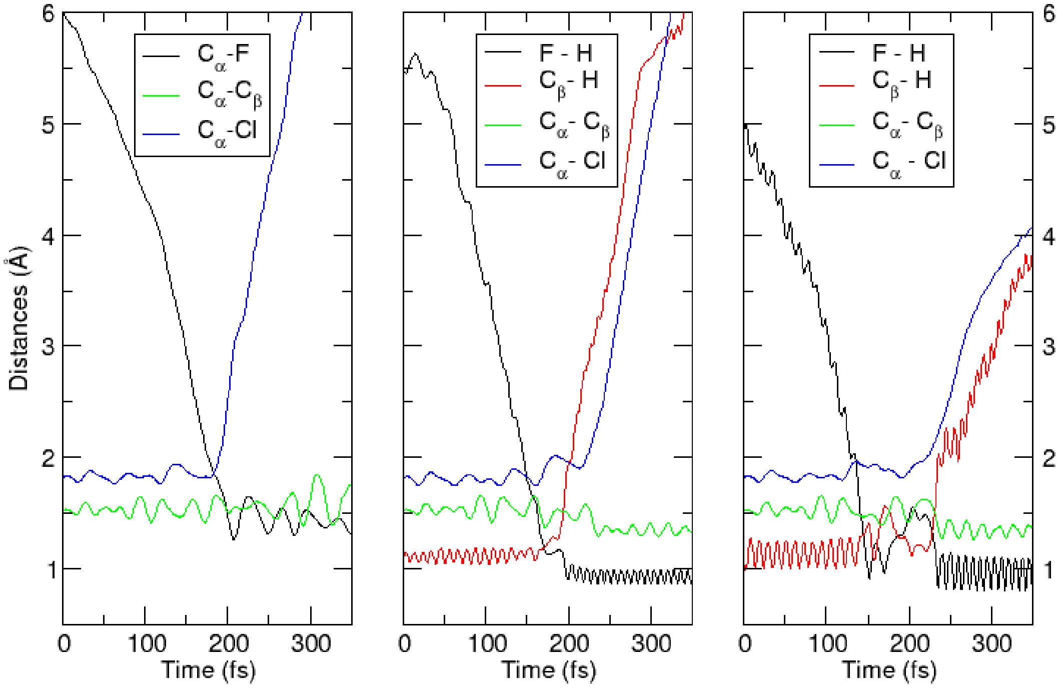

Figure S1: Color plots of the relevant interatomic distances versus time for the reactive trajectories featuring the halogen substitution and base-induced elimination mechanisms of the polyatomic F^-+ CH_3CH_2Cl reaction. Left panel: nucleophilic substitution, S_N2 mechanism. Shown distances are: C_α–F, C_α–C_β and C_α–Cl. Middle panel: based-induced syn-elimination, *syn*–E2 mechanism. Shown distances are: F–H, C_β–H, C_α–C_β, and C_α–Cl. Right Panel: based-induced anti-elimination, *anti*–E2 mechanism. Shown distances are: F–H, C_β–H, C_α–C_β, and C_α–Cl.
