# Supplementary material for: Time-Resolved Chemical Bonding Structure Evolution by Direct-Dynamics Chemical Simulations
Source: J Phys Chem Lett. 2024 Nov 28;15(49):12138–43. doi: 10.1021/acs.jpclett.4c03010 (PMC11648084; doi:10.1021/acs.jpclett.4c03010)
Supplement: Supplementary file 5 — jz4c03010_si_005.pdf [file jz4c03010_si_005.pdf]

jz-2024-030107.R1

Name: Peer Review Information for "Time-resolved chemical bonding structure evolution by direct-dynamics chemical simulations"

First Round of Reviewer Comments

Reviewer: 1

Comments to the Author

The authors analyze how chemical bonds break and form along quasi-classical trajectories using the Global Natural Orbital Functional method. The approach is applied to the F- + CH3CH2Cl SN2/E2 reaction, which system was recently investigated both experimentally and theoretically. I think the present analysis is novel and provides new insights into the mechanism of the F- + CH3CH2Cl reaction. The paper is well written and the SI movies are nicely prepared. Knowing the current interest in SN2/E2 processes, the present study may attract the broad attention of the JPCL readership. Therefore, I recommend publication of this work in JPCL once the authors address the comments given below.

Specific comments:

Ref. 18 should be updated with volume and page numbers.

It is unclear how many trajectories were run for this study. Furthermore, it should also be clarified whether the results are representative. For example, the authors discuss the time frames of the different processes, however, these seem to be based on a single trajectory for each pathway. Are the present results statistically significant?

In Figure 1 Hartrees should be hartrees. Furthermore, it would be more informative to show energies relative to the reactants, maybe in eV or kcal/mol.

The meaning of the "Here we can see that the initial proton abstraction…" sentence is not clear (it seems grammatically incorrect).

"collision energy actives" should be "collision energy activates"

Behind Eq. (7) "." should be ",".

In the reference list, some of the journal names should be given in their shorter forms.

In the SI, Fig. 1 should be Fig. S1 and should be written with larger letters. In Fig. S1 a panel for SN2 would also be appropriate.

Reviewer: 2

Comments to the Author

This manuscript reports trajectory simulations for the reaction of F- with CH3CH2Cl, which may proceed via substitution or elimination pathways. Three trajectories are analyzed for which the initial conditions were chosen such that SN2, syn-E2 and anti-E2 reaction mechanisms were observed. Then the time evolution of selected molecular orbitals were inspected.

The manuscript presents a view on these reactions that probably has a didactic value. However, I do not see relevant new scientific insight that would warrant publication in the Journal of Physical Chemistry Letters. Furthermore, the introduction is written in the style of an undergraduate textbook and no clear conclusions are drawn. I think in the present form this manuscript should not be accepted for publication.

Reviewer: 3

Comments to the Author

In this work, the authors add an examination of the chemical bonding orbitals to a direct-dynamics study of the exemplary F- + CH3CH2Cl reaction, which has three pathways. Overall, I found the study to be insightful and definitely worth publishing; however, I think there are some issues associated with how well the results are described.

I think more explanation of what is being shown in Figure 1 is needed. What are the oscillations shown at short times? The font size is too small for good legibility.

The lack of an adequate explanation of Figure 1 leads then to being unclear. To me, there is a mismatch in what is shown in Figure 1 and the description of the surfaces on page 3, column 1. Here, it says that all reactions have double-well potential energy surfaces and the submerged barriers are extremely close. In Figure 1, it appears as though both E2 reactions have three wells and that the barrier for syn-E2 is higher than for SN2 and anti-E2. Please explain the discrepancies.

Furthermore, later on, when the timing for the reactions is described, it does not match that shown in Figure 1. For example, Figure 1 shows that overcoming the second barrier occurs at basically the same time (about 220 fs) for both E2 reactions, whereas the text says they differ by 40 fs. Please explain.

Oddly, I find that the TOC graphic actually shows the timing much better. Perhaps this graphic should be included in the text.

I also think the authors need to provide a better qualitative explanation of why the orbitals shown progress as they do. For example, in Figure 2, the beginning of the first row looks the same as the end of the second row, so why do they switch. This becomes more important in understanding Figure 3 (syn-E) versus Figure 4 (anti-E2). In figure 3, the beginning of the second row (F-) looks like the end of the third row. In contrast, in Figure 4, the beginning of the third row (again F-) stays in the third row, leading to the “rebound” conclusion, but begs the question of why syn-E2 isn’t also rebound.

I am unable to comment on the Appendix (which is not labeled as such).

Minor stuff:

Page 2, column 2, “thorough” not “thoroughly”

Two lines later, please specify which “previous results” in particular you are referring to by citation.

All modern journals, including this one, recommend the use of IUPAC energy units. As kcal/mol appears only one place, why not replace this with eV units as utilized elsewhere (or kJ/mol).

“data” is plural, thus, it should be “these data” not “this data”.

Page 4, column 1, six lines from the bottom. Remove the second “sequential” in this sentence.

Page 5, column1, five lines from bottom, remove “at”.

Page 5, column 1, bottom, run-on sentence starting “The present study…” “study reports on the study”; “of the F- + CH3CH2Cl” reaction?; and (remove comma) reveals how it”, what does “it” refer to?

Page 5, column 2, second to last sentence. Remove both commas and replace “for” by “because”?

Author's Response to Peer Review Comments:

' Donostia International Physics Center - 20018 Donostia, Spain

 (+34) 943 018328

$ mario.piris@ehu.eus

November 19, 2024
Journal of Physical Chemistry Letters

AMERICAN CHEMICAL SOCIETY

RE : Revision of Manuscript jz-2024-030107

Dear Editor,

Thank you very much for prompt response to our submission with your ref.: Manuscript ID jz-2024-030107. We thank the reviewers for their thoroughly examination of our manuscript. All their comments and suggestion are well taken, and we have revised the manuscript and the supporting information accordingly.

Please find enclosed the revised manuscript and the supporting information, which we believe have both been strengthened, along with our detailed point-by-point responses to all the concerns raised by the reviewers. Notice that our answers are indented just following suit the corresponding reviewer's criticism/suggestion. Below, we address these comments individually.

Reviewer #1 Comments:

The authors analyze how chemical bonds break and form along quasi-classical trajectories using the Global Natural Orbital Functional method. The approach is applied to the $F^{-} + CH_3CH_2Cl$ SN2/E2 reaction, which system was recently investigated both experimentally and theoretically. I think the present analysis is novel and provides new insights into the mechanism of the $F^{-} + CH_3CH_2Cl$ reaction. The paper is well written and the SI movies are nicely prepared. Knowing the current interest in SN2/E2 processes, the present study may attract the broad attention of the JPCL readership. Therefore, I recommend publication of this work in JPCL once the authors address the comments given below.

Specific comments:

- Ref. 18 should be updated with volume and page numbers.

Ref. 18 has been updated with volume and page numbers.

- It is unclear how many trajectories were run for this study. Furthermore, it should also be clarified whether the results are representative. For example, the authors discuss the time frames of the different processes, however, these seem to be based on a single trajectory for each pathway. Are the present results statistically significant?

  The polyatomic $F^- + CH_3CH_2Cl$ reaction, has been extensively studied and has became a "canonical" benchmark type reaction for either calibration and/or subsequent concept-demonstration studies. Our study belongs to the latter class. Particularly relevant for our study is that the range of initial conditions for each of the three major pathways dealt with in our study have already been precisely characterized earlier (see Refs. [11-16]). Consequently, we have chosen accordingly the initial conditions leading to each of these three pathways and, have run our direct-dynamics chemical simulations, implementing an electronic structure method able to describe correctly all types of chemical bonds (see Refs. [5-8]) all along the interatomic distances while they break and form. We have made sure that the analyzed trajectories are representative of the chemical bonding evolution for the major three different mechanisms of the reaction, so that we can deliver reliable new insight about the operating reaction mechanisms, namely: the specific time-ordered sequence in which chemical bonds are made and/or broken.

- In Figure 1 Hartrees should be hartrees. Furthermore, it would be more informative to show energies relative to the reactants, maybe in eV or kcal/mol.

  We have changed Figure 1 in accordance with the reviewer's suggestion, and have rewritten its caption accordingly. The shown potential profiles, in eV, are relative to the reactants, namely, F^- with a translational energy of 0.2 eV and CH_3CH_2Cl in the ground state.

- The meaning of the "Here we can see that the initial proton abstraction…" sentence is not clear (it seems grammatically incorrect).

  We agree, the sentence is confusing. Consequently, we have substituted it by this one: Here we can see the initial proton abstraction from C_β by the incident F^- anion as reflected in the time evolution of the two natural orbitals shown in the bottom rows of Fig. 3, between t = 160 fs and t = 194 fs. Thus, one can observe clearly the formation of …

- "collision energy actives" should be "collision energy activates"

  We have corrected this typographical error.

- Behind Eq. (7) "." should be ",".

  We have corrected this typographical error.

- In the reference list, some of the journal names should be given in their shorter forms.

We have given journal names in their standard short form.

- In the SI, Fig. 1 should be Fig. S1 and should be written with larger letters.

Yes indeed. We have replaced it accordingly.

- In Fig. S1 a panel for S_N2 would also be appropriate.

We have included a panel for the S_N2 mechanism.

Reviewer #2 Comments:

This manuscript reports trajectory simulations for the reaction of F^- with CH_3CH_2Cl, which may proceed via substitution or elimination pathways. Three trajectories are analyzed for which the initial conditions were chosen such that SN2, syn-E2 and anti-E2 reaction mechanisms were observed. Then the time evolution of selected molecular orbitals were inspected.

I do not see relevant new scientific insight that would warrant publication in the Journal of Physical Chemistry Letters … and no clear conclusions are drawn.

We found this to be quite a disconcerting recommendation, because starting from the very second paragraph of the abstract and all along the text till the "Conclusion" section, we have tried hard to put forward that it is just "insight" what the current manuscript delivers. Indeed, we advocate for a fresh view of the direct-dynamics chemical simulations from the point of view of the specific time-evolution of the (purposefully) chosen (natural) orbitals through the reactive trajectories. Since, these natural orbitals have been found (and so documented earlier, see Refs. [5-8]) to be able to provide a chemically accurate meaningful description of the full processes of breaking and making chemical bonds, we claim that they do deliver "insightful" chemical information about how chemical molecules are transformed into another chemical molecules by virtue of breaking some bonds and replacing them by newly formed ones in a well-defined time ordered manner. This is what constitutes the reaction mechanism, one essential piece of chemical information in reactivity studies. We have emphasized that despite the massive amount of information delivered by direct-dynamics chemical simulations, the chemical bonding structure evolution is seldom addressed. But, with the advent of computationally affordable natural orbitals, direct-dynamics chemical simulations can now be made to reliably decipher the chemical bonding structure evolution along reactive trajectories. We have demonstrated, that this is feasible by studying the three major reactive trajectories of the extensively benchmark F^- + CH_3CH_2Cl polyatomic reaction, and have diligently stated our conclusions in the "Abstract", put forward in the "Results and discussion" section, and finally summarized them in a concise manner in the "Conclusions" section. We claim that our conclusions are new and original, and more importantly, they demonstrate that a shift of focus towards analyzing bonding evolution in directdynamics chemical simulations can yield a richer chemical

perspective of reaction mechanisms by identifying the participating components the chemists' way, i.e.: by their chemical bonds.

The manuscript presents a view on these reactions that probably has a didactic value ... Furthermore, the introduction is written in the style of an undergraduate textbook ... I think in the present form this manuscript should not be accepted for publication. Is the paper likely to interest a substantial number of physical chemists, not just specialists working in the authors' area of research?: No

We are aware that the Journal of Physical Chemistry Letters is aimed at *reporting new and original experimental and theoretical basic research of interest to physical chemists, biophysical chemists, chemical physicists, physicists, material scientists, and engineers*. Given such broad audience, both the "didacticity" and a "writing style" accessible to such a diverse audience (undergraduate textbook style is definitely one such style) might be seen as "laudable", in our opinion. We have indeed written the paper is such a way having this in mind.

All in all given that we have drawn attention to a seldom mentioned aspect of direct-dynamics chemical simulations which brings to the fore relevant chemical insight directly informing about the reaction mechanism of reactive processes, and since we have delivered all this information in an accessible and didactic manner we, along with the remaining referees, believe that this manuscript is appropriate for the journal.

Reviewer #3 Comments:

In this work, the authors add an examination of the chemical bonding orbitals to a direct-dynamics study of the exemplary $F^- + CH_3CH_2Cl$ reaction, which has three pathways. Overall, I found the study to be insightful and definitely worth publishing; however, I think there are some issues associated with how well the results are described.

I think more explanation of what is being shown in Figure 1 is needed. What are the oscillations shown at short times? The font size is too small for good legibility.

We have added on page 3, just above the "Results and discussion" section the following paragraph to further clarify the meaning of Figure 1, in accordance with the reviewer's suggestion:

"The fast fluctuations of the total potential energy for the three mentioned pathways, shown in Fig. 1 at both short- and long-times, reflect the energy oscillations of the quasi-harmonic vibrational modes of the reactants and products respectively. Observe that at intermediate times, the oscillation pattern markedly differs from quasi-harmonic, indicative of chemical bonding rearrangement being taking place. The precise nature of the occurring rearrangement for each pathway will be discussed below by inspecting the time evolution of the relevant bonding natural orbitals."

The lack of an adequate explanation of Figure 1 leads then to being unclear. To me, there is a mismatch in what is shown in Figure 1 and the description of the surfaces on page 3, column 1. Here, it says that all reactions have double-well potential energy surfaces and the submerged barriers are extremely close. In Figure 1, it appears as though both E2 reactions have three wells and that the barrier for syn-E2 is higher than for SN2 and anti-E2. Please explain the discrepancies. Furthermore, later on, when the timing for the reactions is described, it does not match that shown in Figure 1. For example, Figure 1 shows that overcoming the second barrier occurs at basically the same time (about 220 fs) for both E2 reactions, whereas the text says they differ by 40 fs. Please explain.

> We have clarified that it is the total potential energy, not the total energies, of the reactive complex that is plotted as a function of time in Figure 1. Consequently, Figure 1 does not provide information about energy barriers. The transition between molecular structures is shown in Figures 2-4 for the S_N2, syn-E2, and anti-E2 pathways, respectively. Figure 1 does not illustrate potential energy surfaces, and specific details regarding potential wells and barriers cannot be derived from it. Our statement about these reactions having double-well potential energy surfaces with closely positioned submerged barriers is based on prior studies, such as Figure 1 in reference 15 of our paper (DOI: 10.1038/s41557-021-00753-8). With the extended explanation given to the meaning of Figure 1, we believe that the concerns raised by the reviewer have now been clarified.

Oddly, I find that the TOC graphic actually shows the timing much better. Perhaps this graphic should be included in the text.

> This might simply be a question of different tastes, but since the paper is aimed at making emphasis on the analysis of the time evolution of the natural orbitals, we insist that the graphics of Figs. 2-4 adhere closer to the spirit of our narrative.

I also think the authors need to provide a better qualitative explanation of why the orbitals shown progress as they do. For example, in Figure 2, the beginning of the first row looks the same as the end of the second row, so why do they switch. This becomes more important in understanding Figure 3 (syn-E) versus Figure 4 (anti-E2). In figure 3, the beginning of the second row (F-) looks like the end of the third row. In contrast, in Figure 4, the beginning of the third row (again F-) stays in the third row, leading to the “rebound” conclusion, but begs the question of why syn-E2 isn’t also rebound.

> We agree with the reviewer. The captions of Figures 2-4 have been modified to explain the sequence of the orbitals’ evolution.
>
> The Caption of Fig. 2 has been complemented with: “The top row natural-orbitals’ snapshots show the adiabatic evolution of the $\sigma(Cl–C_\alpha)$ bond to the $\sigma(C_\alpha–F)$ bond. The bottom row shows the adiabatic evolution of the $F^-(2p_z)$ orbital to the $Cl^-(3p_z)$ orbital. The tiny orange and green dots represent the positions of F^- and Cl^-, respectively.”

The Captions of Figures. 3,4 have been complemented with: “The top row natural-orbitals’ snapshots show the adiabatic evolution of the σ(Cl–C_α) bond to the π(C_α–C_β) bond. The middle row shows the the adiabatic evolution of the F^-(2p_z) orbital to the Cl^-(3p_z) orbital, and the bottom row shows the adiabatic evolution of the σ(H-C_β) bond to the σ(F–H) bond. The tiny orange and green dots represent the positions of F^- and Cl^-, respectively. See text for further details.”

I am unable to comment on the Appendix (which is not labeled as such).

Well spotted. The Appendix has been labeled as such.

Minor stuff:

- Page 2, column 2, “thorough” not “thoroughly”.

  The adverb “thoroughly” was replaced with the adjective “thorough”.

- Two lines later, please specify which “previous results” in particular you are referring to by citation.

  We have specified which previous results we were referring to by citing the works corresponding to references 10 to 18 in the paper.

- All modern journals, including this one, recommend the use of IUPAC energy units. As kcal/mol appears only one place, why not replace this with eV units as utilized elsewhere (or kJ/mol).

  We have replaced the value 30 kcal/mol with 1.3 eV to align with the use of IUPAC energy units.

- “data” is plural, thus, it should be “these data” not “this data”.

  “this data” has been replaced by “these data”.

- Page 4, column 1, six lines from the bottom. Remove the second “sequential” in this sentence.

  The second “sequential” was removed from the sentence.

- Page 5, column 1, five lines from bottom, remove “at”.

  The word “at” was removed from the sentence, so it now reads: “to look for”

- Page 5, column 1, bottom, run-on sentence starting “The present study. . . ” “study reports on the study”; “of the F- + CH3CH2Cl” reaction?; and (remove comma) reveals how it”, what does “it” refer to?

  The sentence beginning with “The present study ...” has been revised to: “The present study reports on the adiabatically relaxed molecular natural orbitals along selected trajectories for the canonical bimolecular (*i*) nucleophilic substitution and (*ii*) base-induced elimination reaction mechanisms of F^- + CH_3CH_2Cl, revealing how these

analyses yield significant chemical insight into both competing reaction mechanisms."

- Page 5, column 2, second to last sentence. Remove both commas and replace "for" by "because"?

The commas have been eliminated, and "for" has been replaced by "because".

We believe that all the points raised by the reviewers have been convincingly discussed and we have followed all their recommendations, so the manuscript has improved notably compared to its previous version and, therefore, we appreciate the input of the reviewers. With the modifications made, as described above, we hope that our manuscript can be accepted for publication in the Journal of Physical Chemistry Letters.

We look forward to your reply.

Thank you for considering our manuscript for publication.

Sincerly,

Prof. PIRIS Mario
